# Anti-stress effects of human placenta extract: possible involvement of the oxidative stress system in rats

**DOI:** 10.1186/s12906-018-2193-x

**Published:** 2018-05-08

**Authors:** Hyun-Jung Park, Hyun Soo Shim, Sunyoung Lee, Dae Hyun Hahm, Hyejung Lee, Chang Taek Oh, Hae Jung Han, Hyi Jeong Ji, Insop Shim

**Affiliations:** 1 Department of Science in Korean Medicine, Graduate School, College of Korean Medicine, Kyung Hee University, Seoul, 02447 South Korea; 2 Business Development Division, Green Cross WellBeing, Seongnam, Gyeonggi-Do 13595 South Korea; 3 Corporate Development Division, Green Cross Corp, Yongin, Gyeonggi-Do 16924 South Korea; 4 Department of Physiology, College of Medicine, Kyung Hee University, Seoul, South Korea; 5 Department of Food Science & Biotechnology, College of Science and Engineering, Kyonggi University, Suwon, South Korea

**Keywords:** Fatigue, Forced swimming test, Glutathione peroxidase (GPx), Human placenta hydrolysate (hPH), Nicotinamide adenine dinucleotide phosphate-diaphorase (NADPH-d), Immobilization stress

## Abstract

**Background:**

Human placenta hydrolysate (hPH) has been utilized to improve menopausal, fatigue, liver function. Its high concentration of bioactive substances is known to produce including antioxidant, anti-inflammatory and anti-nociceptive activities. However, its mechanisms of stress-induced depression remain unknown.

**Methods:**

The present study examined the effect of hPH on stress-induced depressive behaviors and biochemical parameters in rats. hPH (0.02 ml, 0.2 ml or 1 ml/rat) was injected intravenously 30 min before the daily stress session in male Sprague-Dawley rats exposed to repeated immobilization stress (4 h/day for 7 days). The depressive-like behaviors of all groups were measured by elevated plus maze (EPM) and forced swimming test (FST). After the behavior tests, brain samples of all groups were collected for the analysis of glutathione peroxidase (GPx) and nicotinamide adenine dinucleotide phosphate-diaphorase (NADPH-d) staining.

**Results:**

Treatment with hPH produced a significant decrease of immobility time in the FST compared to the controls. Additionally, hPH treatment elicited a slightly decreasing trend in anxiety behavior on the EPM. Furthermore, hPH increased the level of GPx protein in the hippocampus, and decreased the expression of NADPH-d in the paraventricular nucleus (PVN).

**Conclusion:**

This study demonstrated that hPH has anti-stress effects via the regulation of nitric oxide (NO) synthase and antioxidant activity in the brain. These results suggest that hPH may be useful in the treatment of stress-related diseases such as chronic fatigue syndrome.

## Background

Stress is a common and unavoidable phenomenon in life, and excess stress influences mental and physical health. Repeated immobilization stress are activated the hypothalamic-pituitary adrenal (HPA) axis also that results in the release of stress hormone such as catecholamines and catecholamines [1]. High concentration of glucocorticoids show both short and long-term negative changes on emotional and physiological effects [2]. At the behavioral response, repeated stress has been increased depressive behaviors such as anxiety, anhedonia and deficiency of learning and memory [3] and to be a risk factor for psychosomatic or psychiatric illness, such as anxiety [4, 5]. Furthermore, stress is associated with oxidative damage (i.e. free radical damage) [6]. The central nervous system is more vulnerable to free radical damage that induce brain’s high oxygen consumption, relative paucity of antioxidant enzymes and also abundant lipid content [7]. Acute restraint stress stimulates numerous cellular cascades that lead to increased production of reactive oxygen species (ROS) [8] and nitric oxide (NO)-producing neurons regulate the response of the HPA axis to various stress models [9]. Increased physiological stress is also related to the overproduction of reactive oxygen metabolites, and without sufficient ability to clear those metabolites, can have direct deleterious effects on tissues and ultimately survival [10] . On the other hand, decreased activities of antioxidant enzymes dismutase and such as glutathione peroxidase (GPx) and superoxide dismutase have been reported in rodents submitted to restraint stress [11]. Recently, many studies proved the antidepressant effect of compounds or materials possessing antioxidant-like properties and they may be of interest as a therapeutic agent for the treatment of depressive disorders [12–15].

Human placenta hydrolysate (hPH) is known to consist of amino acids, nucleic acids, minerals and unknown effective materials [16, 17]. hPH was used for wound healing or immune enhancement where it exerted its therapeutic activities via the regulation of NO production [18–20]. hPH was usually used for treatment of various diseases such as chronic liver diseases, menopausal syndrome, chronic fatigue, and skin pigment disease [21]. In addition, human placental extract ameliorates structural lung changes [22] and protect cartilage degradation [23] and has anti-inflammatory and analgesic effect [24] and pain relief [25] in animal test. Tohoku et al. reported that subchronic administration of placental extract had the effect of increasing all the monoamines and decreasing the monoamine oxidase (MAO) activity [26]. Also, placental extract improves hippocampal neuronal loss and fear memory impairment resulting from chronic restraint stress in ovariectomized mice [27]. Moon et al. also reported anti-fatigue effect of porcine placenta on mice [28]. Another study showed that human placental extract are associated with elevated maternal pain threshold to noxious stimuli [25]. However, its mechanisms of stress-induced depression in animal model remain unknown. In the present study, we focused on the biological effects of hPH on stress induced depressive rats.

However, animal model studies on hPH are insufficient to scientifically demonstrate its working mechanisms.

In this study, we aimed to investigate whether hPH could modulate depressive–like behavior and production of oxidative stress markers. To achieve this goal, hPH’s antidepressant effect was tested via an elevated plus maze (EPM) and forced swimming test (FST). Moreover, the influence of hPH on the levels of NADPH-d and GPx was further assessed in the brain regions using the enzyme linked immunosorbent assay (ELISA) and immunohistochemistry (IHC).

## Methods

### Animals

Male Sprague-Dawley (SD) rats weighing 250–280 g were purchased from Samtako Inc. (Osan, Korea). The animals were acclimated for at least 1 week prior to the experimentation. They were housed in individual cages under 12/12-h light/dark cycle and at room temperature. Food and water were made available ad libitum. Experiments were approved by the Kyung Hee University Institutional Animal Care and Use Committee (KHUASP (SE)-13–014) and the experimental protocol was also approved by the Institutional Review Committee for the use of Human or Animal Subjects.

### Preparation of hPH

Human placenta hydrolysate (hPH) is prepared with human placenta (including umbilical cord). The placenta is acquired free of charge at the obstetrics and gynecology which is contracted with Green Cross WellBeing in Korea, along with a consent from donation from donor. The hydrolysate of human placenta is manufactured by chemical process with HCl and pepsin, followed by dialysis, heat treatment and hydrolysis. hPH consists of various amino acids including leucine (0.12%), arginine (0.08%), alanine (0.08%), phenylalanine (0.08%), serine (0.07%), threonine (0.06%), valine (0.04%), tyrosine (0.03%), methionine (0.03%), lysine (0.1%). Insoluble macromolecules, such as polysaccharides, polynucleotides, etc. were excluded during the manufacturing process.

### Stress procedures

SD male rats were exposed to repeated immobilization stress (4 h/day for 7 days).

hPH used in this study (Laennec, Green Cross WellBeing) was manufactured by Green Cross Corp. through the hydrolysis of human placenta with HCl and pepsin. hPH (0.02 ml, 0.2 ml or 1 ml/rat) was injected intravenously 30 min before the daily stress session in hPH groups, while control groups were given sterile saline. Rats were randomly divided into five groups: the naïve normal group (normal, *N* = 10), the stressed and saline treated group (control, *N* = 6), the stressed and hPH 0.02 ml treated group (hPH 0.02, *N* = 7), the stressed and hPH 0.2 ml treated group (hPH 0.2, *N* = 8) and, the stressed and hPH 1 ml treated group (hPH 1, N = 6). As a positive control for the purpose of comparison, fluoxetine was dissolved in saline and administered orally (30 mg/kg) daily for 1 week.

### Forced swimming test (FST)

FST is usually used tests for assessing antidepressant activity. The transparent Plexiglas cylinder (50 cm deep, 20 cm diameter) filled with 26 °C water to a depth of 30 cm at room temperature. The rats were tested in the cylinder for 15 min, 24 h prior to the 5-min swimming test. The following behaviors were recorded by trained two observers. Climbing behavior was defined as vertical motion of the forepaws along the side of the wall. Swimming behavior was defined as movement throughout the swim cylinder. Immobility time was defined when the rat made no further attempts to move four paws [29].

### Elevated plus maze (EPM)

After exposure to stress, animals were immediately subjected to EPM tests. The apparatus used in the present study consisted of two closed arms (50 × 10 × 40 cm each), two open arms (50 × 10 cm each), and a central platform (10 × 10 cm), arranged such that the open arms and closed arms were directly opposite each other. The EPM apparatus was constructed from black Plexiglas. Animals were placed in the center and then the parameters were recorded during the 5-min. The time spent on the open arms and the closed arms of the maze was video-taped and recorded for 5 min by S-MART program (Pan-Lab, Barcelona, Spain).

### Anesthetic and euthanasia

After the behavioral tests, rats were anesthetized with sodium pentobarbital (80 mg/kg) intraperitoneally.

### Glutathione peroxidase (GPx) measurement

(ELISA development system, TX, USA). Brain tissue protein was extracted using by PRO-PREP™ solution. The tissue samples were centrifuged at 20,000×g for 10 min at 4∘C. The supernatant was transferred to another tube and was analyzed by ELISA kit Samples (200 ul) and standards were transferred to GPx antibody-coated 96 well plates and assay cocktail (150 ul) was added into all wells. After 10 min incubation, the optical density at 405–414 nm was determined 6 times for 30 min using a microplate reader (Bio-Rad 680, CA, USA). Sample values were calculated from a standard curve.

### NADPH-d staining

After the behavioral testing was completed, then rats were perfused 1X PBS perfusate containing heparin, followed by 800 ml of 4% paraformaldehyde in 0.1 M PBS. The brains were placed fixed brain in 20% sucrose solution and incubate at 4 °C overnight. The brains were cut through the midline with a fresh blade at the level of the PVN. Brain tissue were incubated for 30 min in 0.1 mg/ml nitroblue tetrazolium (Sigma, MO, USA) and 0.1 mg/ml β-nicotinamide adenine dinucleotide phosphate (NADPH) (Sigma, MO, USA) in 0.05 M PBST at 37 °C. And then, the sections were rinsed three times (10 min each) in 0.05 M PBS. The slides were air-dried and then coverslipped with Permount™ solution (Fisher Scientific, CA, USA). The number of NADPH-d positive cells were measured from areas of 200 um^2^ according to the atlas of Paxinos and Watson [30] under the light microscope (DP-20, Olympus, CA, USA).

### Data analysis

All the results are expressed as mean and standard error (SEM). One-way analysis of variance (ANOVA) was performed using SPSS 15.0 software (SPSS Inc., IL, USA) followed by the post hoc least significant difference (LSD) test. An unpaired t-test was performed to determine statistical significance for fluoxetine versus saline comparison. *P* values < 0.05 were considered statistically significant.

## Results

### Forced swimming test

**Fig. 1:**
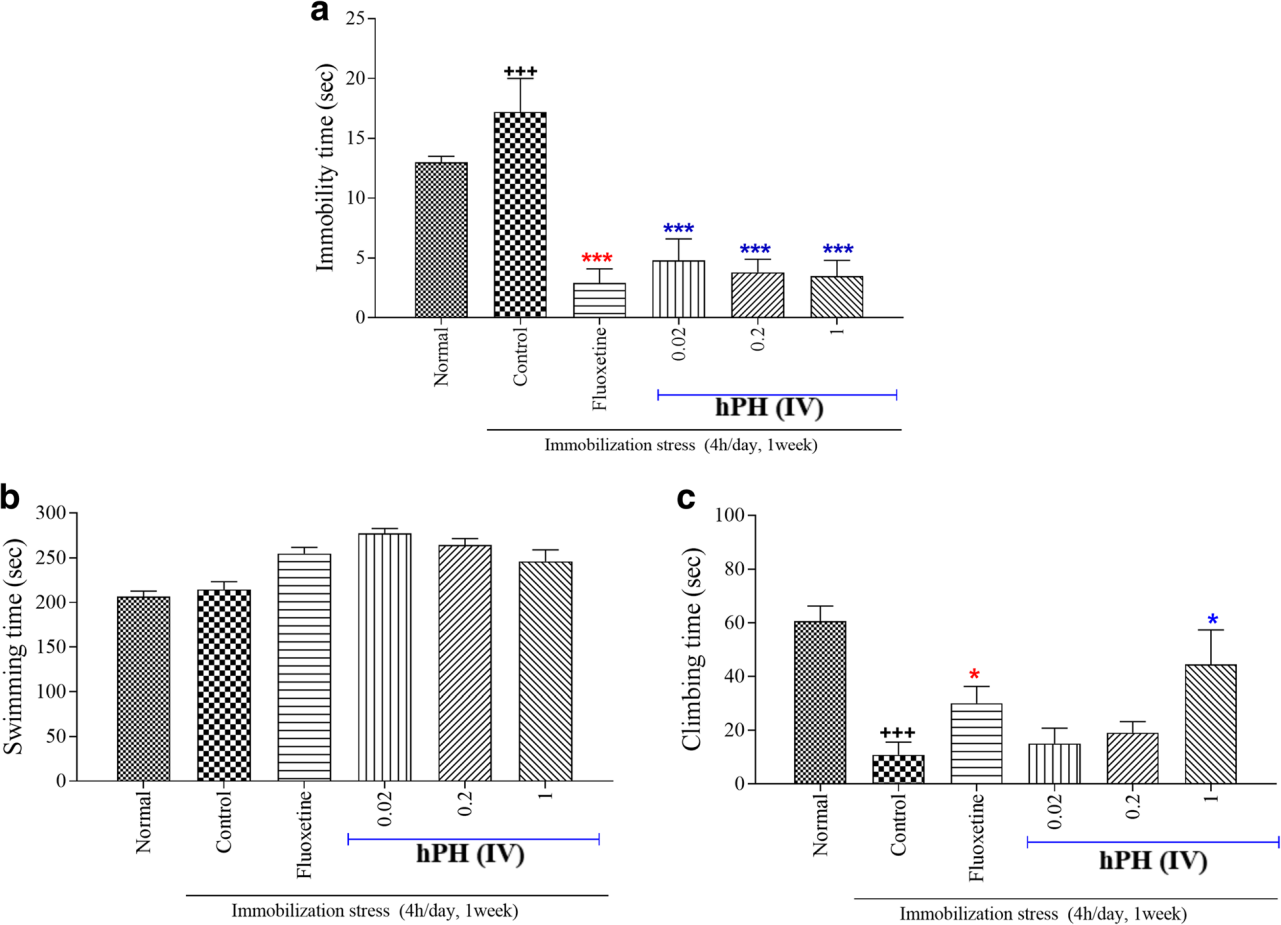
The effects of hPH and fluoxetine treatment on FST. **a** Immobility time, (**b**) Swimming time, and (**c**) Climbing time. Each value represents the mean + S.E.M. ^+++^*P* < 0.001 compared to the normal group and ^*^P < 0.05 and ^***^P < 0.001 compared to the control group

In the forced-swimming test (FST), rats were tested to avoid negative situation (learned helplessness, Fig. 1a-c). Animals displaying increased helplessness, which is a sign of depressive-like behavior. In the FST, the control groups showed longer immobility time than the normal group (Fig. 1a, p < 0.001). However, the immobility time was significantly decreased in hPH treated groups, compared to the control group (Fig. 1a, p < 0.001). The swimming time was not significant difference among groups (Fig. 1b). In addition, the active behavior (climbing) was increased in the hPH 1 ml treated group compared to the control group (Fig. 1c, p < 0.05).

### Elevated plus maze

**Fig. 2:**
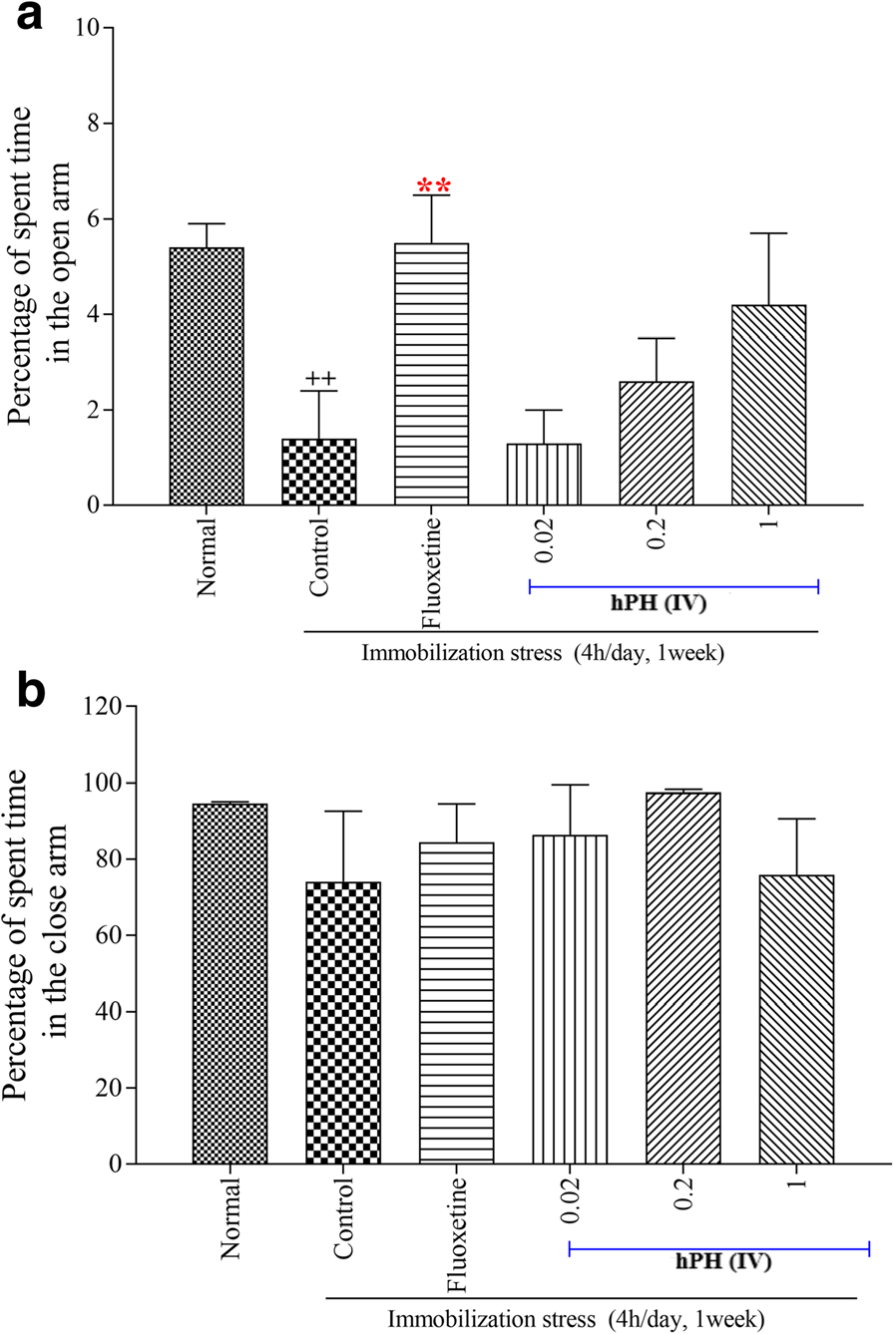
The effects of hPH on EPM. **a** percentage of spent time in the open arm and (**b**) percentage of spent time in the close arm. Each value represents the mean + S.E.M. ^++^*P* < 0.01 compared to the normal group; ^**^P < 0.01 compared to the control group

As shown in Fig. 2, the one-way ANOVA revealed a significant difference between groups regarding the time spent in the open arms or close arms. The control group significantly decreased the time spent in the open arms and increased time in the closed arms compared to that of the normal group. Fluoxetine treatment showed a significantly longer period of time spent in the open arms than that of the control group (*p* < 0.01). Although it was not significant, treatment with hPH also produced a decreasing trend in anxiety behavior on the EPM.

### Glutathione peroxidase (GPx) measurement

**Fig. 3:**
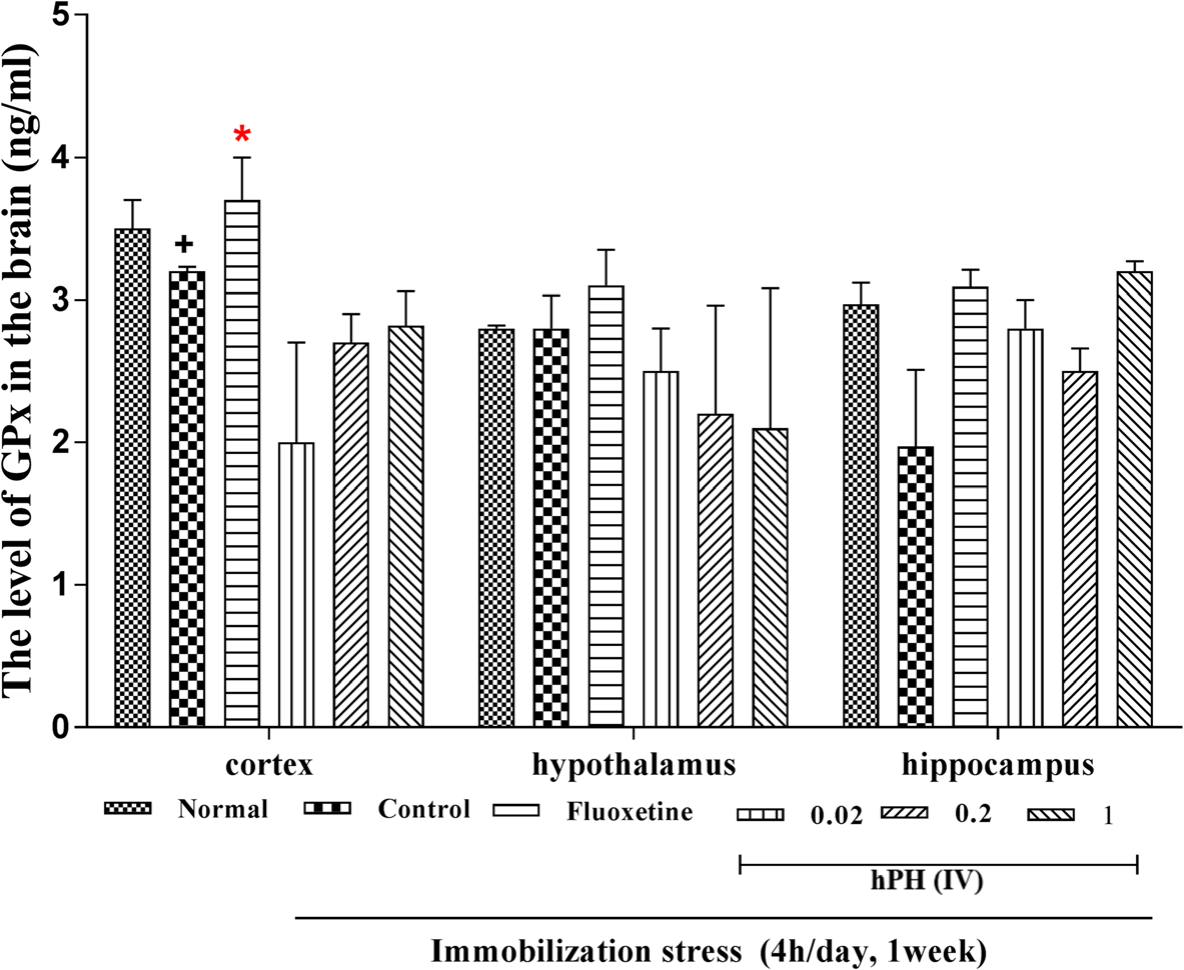
The effects of hPH treatment on the level of GPx in the brain. Each value represents the mean + S.E.M. ^+^*P* < 0.05 compared to the normal group; ^*^P < 0.05 compared to the control group

GPx levels in the cortex, hypothalamus and hippocampus were changed after repeated stress (Fig. 3). hPH or fluoxetine treated group showed a trend of higher GPx levels in the hippocampus when compared to the control group. In contrast, GPx levels within the cortex and hypothalamus were not affected by the treatment of hPH.

### NADPH-d staining

**Fig. 4:**
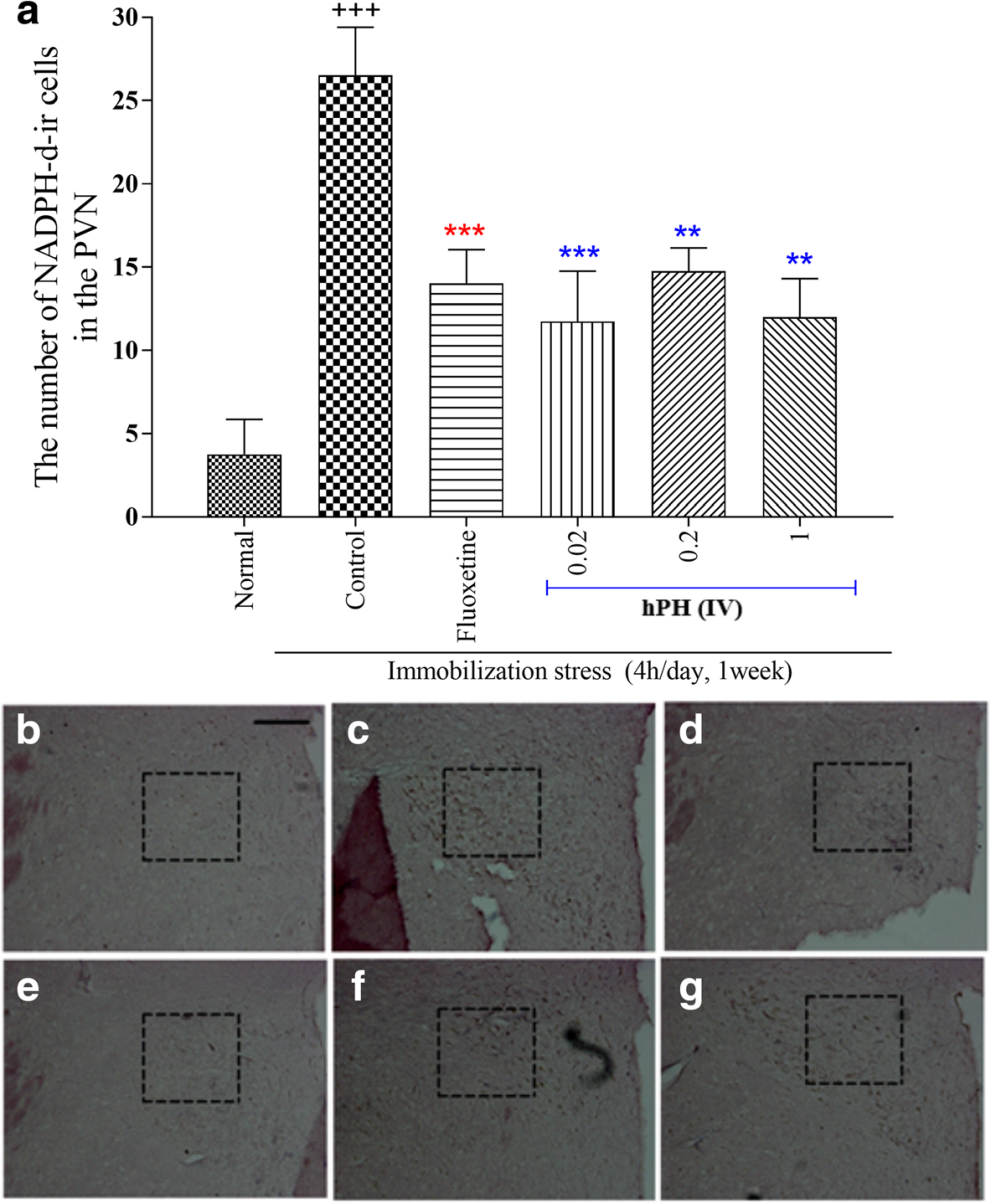
**a** The effects of hPH and fluoxetine treatment on the expression of NADPH-d immunoreactive neurons in the PVN of the each treated group. **b** - **g** Immunohistochemical staining of NADPH-d in the PVN obtained from the each treatment group. **b** Normal, (**c**) Control, (**d**) Fluoxetine, (**e**) hPH 0.02, (**f**) hPH 0.2, and (**g**) hPH 1, respectively. Each value represents the mean + S.E.M. ^+++^*P* < 0.001 compared to the normal group and ^**^P < 0.01, ^***^P < 0.001 compared to the control group

The results of the number of the NADPH-d-positive neurons are shown in Fig. 4a-g. The expression of NADPH-d-positive neurons in the paraventricular nucleus (PVN) of the control group was significantly increased by the repeated immobilization stress, as compared to that of the normal group (*p* < 0.001). However, the expression of NADPH-d-positive neurons in the hPH treated group was significantly lower than that of the control group (*p* < 0.01).

## Discussion

This study proved that the treatment with hPH produced significant decreases in the despair behavior in the FST compared to the control group. The hPH treated groups showed markedly reduced expression of the NADPH-d in the PVN compared to the control group. In addition, the repeated treatment of hPH showed an increasing trend of GPx levels in the hippocampus when compared to the control group. These results suggested that the effects of the repeated treatment of hPH are mediated by the regulation of a repeated stress-induced depressive behavior and the oxidative stress system in the brain.

In a pilot study, we compared the effects of various routes of administration on the immobility in FST using intravenous, intramuscular or subcutaneous injections of hPH (0.002, 0.02, 0.2, 1 and 2 ml/rat). It was found that the most effective route to reduce immobility was intravenous injection with ranges of 0.2 to 2 ml (data not shown). Therefore, we used intravenous injections of hPH at the doses of 0.02, 0.2 and 1 mL/rat in this study.

Chronic stress induces oxidative stress via hyper-activation of the hypothalamic pituitary adrenal (HPA) axis, leading to oversecretion of corticosterone [31]. Moreover, the oversecretion of corticosterone induces depressive-like behaviors such as anxiety and despair. The present study showed that the repeated immobilization stress increased the despair behavior in the FST which was reduced by treatment with hPH or fluoxetine. Recently, some studies reported that repeated restraint stress induced not only depressive behavior but also oxidative stress in the brain region [32–37]. The brain is more susceptible to oxidative stress due to its relatively high consumption of oxygen, high iron content, high fatty acid peroxidation, and low antioxidant capacity compared to other organs [32–34, 36]. Nitric oxide (NO) is the major oxidative stress marker for cellular component’s damage [35, 37–40] and repeated stress increases the NO levels in the brain [9, 41, 42]. Our result also showed that repeatedly stressed rats have significantly increased levels of NADPH-d expression in the PVN. NADPH oxidase has been studied as a major source of reactive oxygen species (ROS) generation in mammalian cells. Sanchez et al. reported that the swim stress enhances the NADPH-d histochemical staining in the PVN of the hypothalamus [43]. In mice, the repeated stress mediates depressive behavior through the upregulation of NADPH oxidase and the resultant metabolic oxidative stress, and the treatment of NADPH oxidase inhibitor apocynin during the stress or post-stress period reduced stress induced responses [8]. In the present study, hPH decreased the expression of NADPH-d in the PVN, suggesting that the inhibition of NADPH oxidase may provide beneficial anti-stress effects. Among important changes in the brain of stress-induced animal models, accumulation of oxidative stress has been noted [8, 44–46]. GPx, an antioxidant enzyme, is a defense system against oxidative stress [47–49], and many antioxidants and free radical scavenging enzyme systems exist in the cell to protect it against the damaging effects of free radicals produced as a part of normal cell respiration and other cellular processes [50]. However, repeated stress affected the level of the antioxidant components as superoxide dismutase, GPx, catalase in the central and peripheral nervous systems [51–54]. Consistent with previous studies, our results showed that the repeatedly stressed rats showed a decreasing trend of GPx levels in the brain regions. Moreover, the repeated treatment of hPH or fluoxetine showed a trend toward increased GPx levels in the hippocampus of the repeatedly stressed rats. Other studies also reported that fluoxetine, one of antidepressants, reduced the amounts of free oxygen radicals [55], catalase levels, and lipid peroxidation [56]. Some studies showed that the human placenta extract and their components have neuro-protective effects by regulating antioxidant actions [57–60].

Taken together, repeated treatment of hPH has an antioxidant effect and HPA axis modulatory activity that could be the important therapeutic strategy for repeated stress-induced behavioral and biochemical changes. These results suggest that hPH may play an active role in the treatment of stress-related responses via regulation of oxidative stress.

## Conclusions

This study demonstrated that hPH has anti-stress effects via the regulation of nitric oxide (NO) synthase and antioxidant activity in the brain. These results suggest that hPH may be useful in the treatment of stress-related diseases such as chronic fatigue syndrome.
